# Obituary

**Published:** 1872-12

**Authors:** 


### OBITUARY.

DIED, on the morning of the 12th inst., near Milledgeville, Dr. CHARLES H. BASS, in the 43d year of his age.

Our brother has gone. Laying aside the muddy vestures of decay he has assumed the spotless robes of immortality. From a world of pain he has passed into the boundless realms of never-ending peace. His soul has found a lodgment in the mansions of his Father's house, where faith reaps its perfect fruition. Death laid no rude or violent hands upon our brother, but touched him with a gentle touch months and months before he claimed his earthly frame. Day by day the vital spark grew imperceptibly dimmer unto its final extinguishment. For the privilege of a gradual decline, with unimpaired mental faculties to the last, death demanded keen physical suffering. Conscious of an eternal reward beyond the grave this suffering was borne without complaint. "He that endureth shall all things inherit," and we feel the assurance of a perfect inheritance for him, for he endured patiently to the end.

A consistent member of the Methodist Church for 27 years —a man full of integrity in all his dealings with his fellow man—modest to a fault, a devoted husband, a loving father, possessed of a well balanced mind, rich in culture and in thought, and with a heart abounding in sympathy for the sick and the distressed. And further: with a characteristic pre-eminent above all other traits his life was enriched, and rendered peculiarly sweet to all that knew him well, by a heart pure and undefiled, to the possessors of which it is promised "they shall see God." C.

The writer deeply laments the untimely death—to human ken—of his old friend and class-mate, Dr. Chas. H. Bass, whose obituary is given above. Dr. Bass was the brother-in-law of the Senior Editor of this journal. A purer, nobler soul rarely dwelt in an earthly tabernacle. At his death, he was Assistant Physician to the State Lunatic Asylum, a position he held for a number of years with great credit to himself and that institution. Our friend has left us! He has gone to a better "country;" a "city" made glorious by the brightness of the "Lamb of God!" W. T. G.

DIED—At his residence in Timberville, Rockingham county, Va., on Tuesday, Oct. 19th, 1872, after a very brief illness, DR. JOHN C. HOMAN, in the 41st year of his age. J. A. A.

